# Two new species of *Chlorospatha* section *Orientales* (Araceae) from western Andes in Colombia

**DOI:** 10.3897/phytokeys.135.38050

**Published:** 2019-11-06

**Authors:** Allison Muñoz-Castillo, Leonardo Guevara-Ibarra, Laura Clavijo, Alejandro Zuluaga

**Affiliations:** 1 Departamento de Biología, Facultad de Ciencias, Universidad del Valle, Calle 13 # 100-00, Cali, Colombia; 2 Grupo de Investigación Ecología y Diversidad Vegetal, Universidad del Valle, Cali, Colombia; 3 Jardín Botánico Joaquín Antonio Uribe de Medellín, calle 73 # 51D-14, Medellín, Colombia

**Keywords:** Araceae, Chlorospatha, new species, taxonomy, tropical Andes, section Orientales

## Abstract

Two new species of Chlorospatha (section Orientales) from the western slope of the Cordillera Occidental in the departments of Valle del Cauca and Choco (Colombia) are described here. The new species represent the first records of section Orientales for Colombia, which was previously known only from the eastern Andes in Ecuador. The two new species are similar to *C. longipoda*, *C. hannoniae* and *C. boosii*. *Chlorospatha minima***sp. nov**. is differentiated by its small overall size (less than 30 cm tall), blade strongly inequilateral with smooth adaxial surface, and spadix less than 2.2 cm long. *Chlorospatha silverstonei***sp. nov**. is differentiated by its large overall size (30–60 cm tall), 1–3 leaves per plant, and quilted adaxial blade surface.

## Introduction

The tribe Caladieae (Araceae) comprises 11 genera and 326 species restricted to Tropical America (Grayum 1986, Cusimano et al. 2011, Boyce and Croat 2011) and 8 species (genus *Hapaline* Schott) in Tropical Asia. The center of diversity of the tribe is Colombia with about 35% of the species, most of them endemic (Grayum 1986, Croat and Hannon 2015, Croat et al. 2017). The genus *Chlorospatha* Engl., with 68 species (Croat and Hannon 2015), is the second largest in the tribe after *Xanthosoma* Schott with 201 species (Boyce and Croat 2011). Morphological characters distinguishing these two genera and others in the tribe have been conflictive (Mayo and Bogner 1988), and the phylogenetic relationships within Caladieae need to be further studied. Cusimano and collaborators (2011) included in their phylogeny only one species of *Chlorospatha* and recovered it as sister of *Xanthosoma*; however, Gonçalves (personal communication) found some species of *Chlorospatha* nested within *Xanthosoma*, using molecular data.

Until 1981, and for almost 100 years, the genus *Chlorospatha* was monotypic with *C. kolbii* Engl. as the only species described. Madison (1981) combined the genus *Caladiopsis* Engl. with *Chlorospatha*, transferred two species from *Caladium* and *Xanthosoma* into *Chlorospatha*, and published three new species in the genus (Croat and Hannon 2015). For the next 20 years, only six new species were described until Croat and Hannon (2004) published a revision of *Chlorospatha* of Antioquia, Colombia, and in 2015, a comprehensive taxonomic treatment that included the description of 39 new species, 19 from Colombia and 12 from Ecuador, one new subspecies, and four unnamed taxa. For a complete history of the taxonomy of the genus *Chlorospatha* see Croat and Hannon (2015).

Most species of *Chlorospatha* have narrow distribution ranges; furthermore, the level of endemism in *Chlorospatha* is the highest among the genera of Araceae in Colombia, with 43 (63%) species endemic to the country (vs. 23 [33%] endemic to Ecuador). Despite the large number of species in Colombia, fewer collections were available compared with Ecuador, highlighting the need for more botanical exploration in the country and the potential for the discovery of several new species (Croat and Hannon 2015). Croat and Hannon (2015) listed 370 collections of *Chlorospatha* from herbaria across the world, but they probably studied more since in the manuscript they mentioned that there are 226 collections from Ecuador, 55 from Central America, and 122 from Colombia. The collection and study, in the past three years, of ca. 70 new collections of *Chlorospatha* from Colombia resulted in the discovery of at least four new species; here we name and describe two new species of *Chlorospatha* from the western slopes of the Colombian Andes. The two new species belong to section Orientales which presents a style not expanded into a mantle, sessile stigma, and was previously, known only from the eastern slopes of the Ecuadorian Andes.

## Materials and methods

We assembled a database with all collections from Croat and Hannon (2015), the TROPICOS database, and the Colombian herbaria COL, CUVC and COAH. Additionally, we reviewed all the collections missing in the most recent revision of the genus (Croat and Hannon 2015) and, between 2012 and 2018, carried out eight expeditions to Serranía de los Paraguas and six expeditions to the Anchicaya river basin, where we collected the two new species. We follow Croat and Hannon (2015) for terminology and its use in the descriptions. All measurements were made from dried herbarium material unless otherwise mentioned.

## Results

Our database comprised, in total, 572 collections of *Chlorospatha*, representing 70 species (including the two described here), with 214 collections from Colombia, seven of them belonging to the two new species. The number of collections per species was very low, with 22 species known only from the type collection, 13 only from two, 27 from less than 10, and merely eight species known from more than 10 collections.

### Taxonomic description

#### Chlorospatha minima

Taxon classificationPlantaeAlismatalesAraceae

Zuluaga & Muñoz-Castillo sp. nov.

34B42FEF-6694-50B4-B8E8-89C8A5B84E97

urn:lsid:ipni.org:names:77202843-1

Figs 1 , 2 , 3 , 4

##### Type.

COLOMBIA. Valle del Cauca: municipio Dagua, corregimiento El Queremal, old road Cali-Buenaventura, 6 km from El Queremal, 3°33'45.6"N, 76°45'27.1"W, 1050–1100 m, 20 May 2017, A. Zuluaga, L. Guevara, M. Llano & A. Muñoz 1645 (**holotype**: CUVC!; **isotypes**: COL!, MO!)

##### Diagnosis.

*Chlorospatha minima* can be distinguished from other species in section Orientales by its overall small size (less than 30 cm tall), smooth adaxial leaf surface, 1–2 inflorescences per axil, and spadix 20.4–22.8 mm long. Additionally, it differs from *C. silverstonei* sp. nov., the other species of this section in the western slopes of the Andes, by having three collective veins (vs. two in C. *silverstonei* sp. nov.), and the primary lateral (secondary) and minor veins glabrous on the abaxial surface (vs. scale-like indument).

Terrestrial herb, 10–25(–30) cm tall; stem subterranean, decumbent, with cataphylls quickly deciduous; internodes 6.6–7.4 × 5.4–7.8 mm, drying matte, dark brown; cataphylls brownish green, 5.2–6.2 cm long, acuminate at apex, drying matte, reddish brown. Leaves 2 to 5, erect-spreading; petioles 8.3–28.2 cm long, fleshy, glabrous, semiglossy, green with faint darker transverse markings, drying matte, dark brown, sheathed 5.2–9.4(–12.0) cm or (1/5–)1/3–1/2 of its total length, rarely more than 1/2; sheath decurrent onto the petiole apex; free portion of the petiole 0.6–3.7 mm diam. midway; blades broadly triangular-ovate, inequilateral, 5.8–14.2(–16.3) × 2.6–10.1 cm, 1.5 to 2.2 times longer than wide, weakly hastate at base, acuminate at apex, usually slightly broader across anterior lobe than at base, not constricted at petiole insertion, glabrous, conspicuously discolor, distance tip to tip across posterior lobes 2.2–9.2 cm wide; both surfaces smooth, glossy, drying semiglossy; abaxial surface with several layers of cells forming a reticulum, 0.3 to 0.4 mm diam.; anterior lobe 5.1–11.9 × 2.6–10.1 cm, 1 to 2 times longer than wide, 2.2 to 5.7 times longer than posterior lobe, wider near petiole insertion, rarely asymmetrical; posterior lobes directed toward base, 0.9–4.6 (–5.2) × 0.8–4.4 cm, 0.7 to 1.4 times longer than wide, narrowly rounded to obtuse at apex, slightly broader at petiole insertion, ± symmetrical, sinus parabolic to spathulate; midrib and major venation usually darker than the surface, round-raised and drying ± flattened abaxially; primary lateral veins 3, rarely 4, per side, arising at 30°–60°, rarely 70°, straight to weakly curving towards the margin; secondary veins abaxially sunken, drying visible and darker than the surface, the primary lateral and minor veins glabrous on the abaxial surface; 3 collective veins that originate from first, second and third basiscopic veins, respectively, ± parallel to margin; basal veins coalescent into a prominent posterior rib, 1–2(–3) acroscopic, 2–3(–4) basiscopic veins; minor veins slightly visible abaxially. Inflorescences erect, 1 to 2 per axil; cataphylls of inflorescence not visible outside the sheath; peduncle held within the sheath, 34.0–51.5(–76.0) × 0.4–1.2 mm, drying dark brown to black; spathe erect (all measurements for the spathe and spadix made from spirit material), 27.0–29.8 mm long, apiculate at apex, 6.6–7.0 mm (1.1 to 1.3 times) longer than spadix; spathe tube green or pale green on outer surface, rarely maroon-tinged, 10.0–12.9 × 4.2–4.3 mm, drying dark brown to black on outer surface; spathe blade maroon-tinged, with green veins on outer surface, green on inner surface, drying dark brown or black, ca. 16.9 mm long, erect after anthesis, then marcescent; spadix erect, 20.4–22.8 mm long, sessile, adnate basally to the spathe for 2.3–3.1 mm (1/5 to 1/3 of the length of pistillate portion); pistillate portion light green, 8.3–8.6 × 2.0 mm; pistils ca. 1.3 mm diam.; stigma light green, sessile, ca. 0.4 mm diam.; fertile staminate portion white, 11.2–12.9 × 2.8–3.0 mm, cylindrical, rounded at apex, drying whitish brown; synandria ca. 1.3 mm diam., coherent; sterile portion white, 2.2–2.5 × 2.7 mm, wider at apex, drying whitish brown; sterile flowers with straight borders, 1.2 mm diam. (viewed from above). Infructescence (measurements made from spirit material) erect or pendent, brown, ca. 25.5 × 8.0 mm, drying mate, dark brown on outer surface; berries green, 2.3–3.9 mm diam.; seeds white, (6–)20 to 24 per berry, 1.2–1.5 × 0.7–0.9 mm, ovoid to ellipsoid, longitudinally striate, minutely white-strophiolate.

##### Etimology.

The epithet *minima* refers to the small size of this species, less than 30 cm tall, the smallest in the genus.

##### Distribution and ecology.

**Figure 1.**
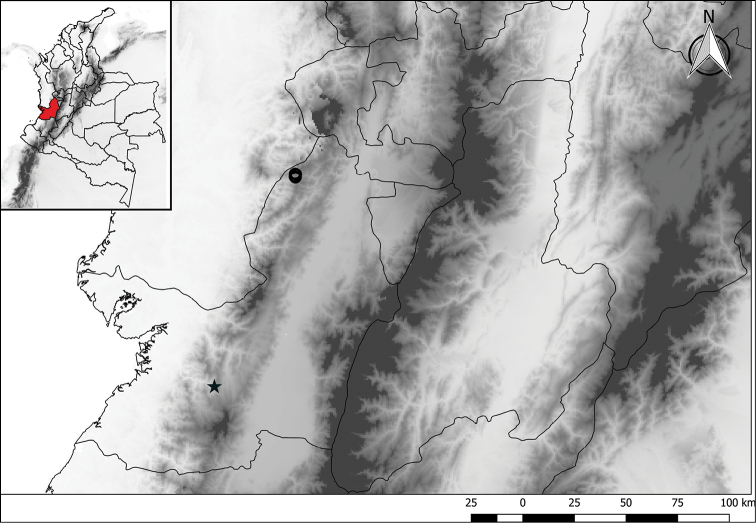
Distribution map of *Chlorospatha minima* (star) and *C. silverstonei* (circle).

*Chlorospatha minima* is endemic to the western slopes of the Colombian Andes in the department of Valle del Cauca. It has been found only in one locality on the old road Cali-Buenaventura at 1000 m, inhabiting humid forest and growing close to a waterfall (Fig. 1).

##### Conservation status.

Despite more than four years of extensive field work, *Chlorospatha minima* is known only from one population located outside the Farallones de Cali National Natural Park, in an area frequently visited by tourists. Because the estimated extent of occurrence is less than 100 km^2^, the only population known has less than 100 individuals, and the quality of habitat is declining, *C. minima* could be assessed as Critically Endangered, according to the IUCN criteria (IUCN 2012, 2017).

##### Discussion.

**Figure 2.**
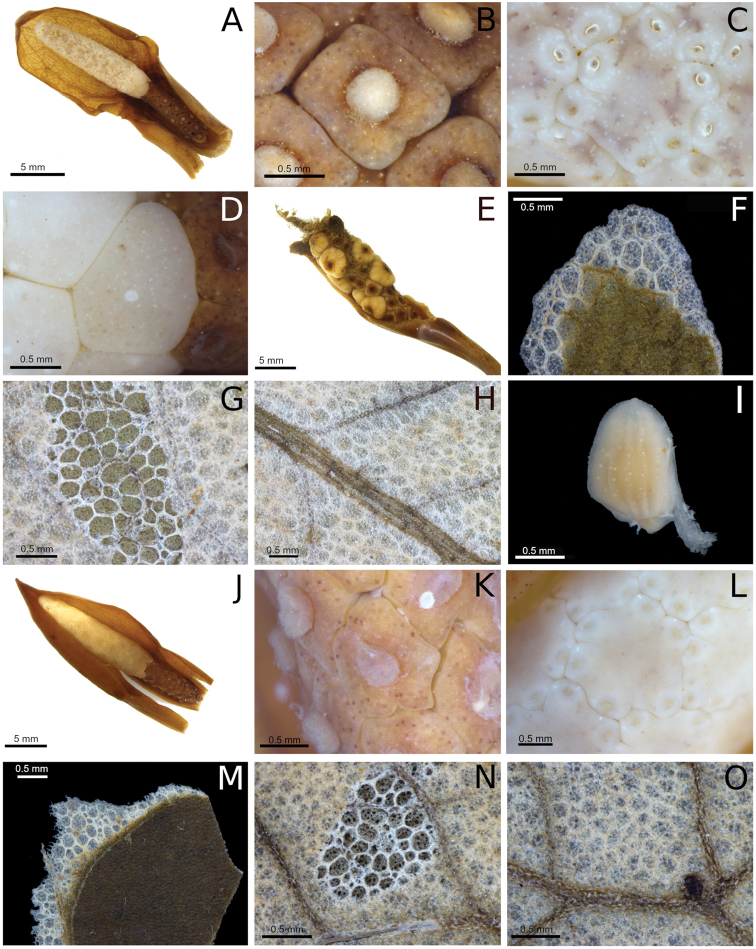
Inflorescence and leaf characters of *Chlorospatha minima* and *C. silverstonei*. **A–I***C. minima***J–O***C. silverstonei***A** inflorescence **B** female flowers **C** male flowers **D** sterile flowers **E** infructescence **F** adaxial blade surface, note the reticulum of cells in the peeling of abaxial surface **G** abaxial blade surface **H** primary lateral and minor veins glabrous on abaxial surface **I** seed **J** inflorescence **K** female flowers **L** male flowers **M** adaxial blade surface, note the layers of cells **N** abaxial blade surface **O** primary lateral and minor veins with scale-like indumentum on abaxial surface. (Photographs by Juan Felipe Ortega and the imaging laboratory at the Biology graduate program at Universidad del Valle).

**Figure 3.**
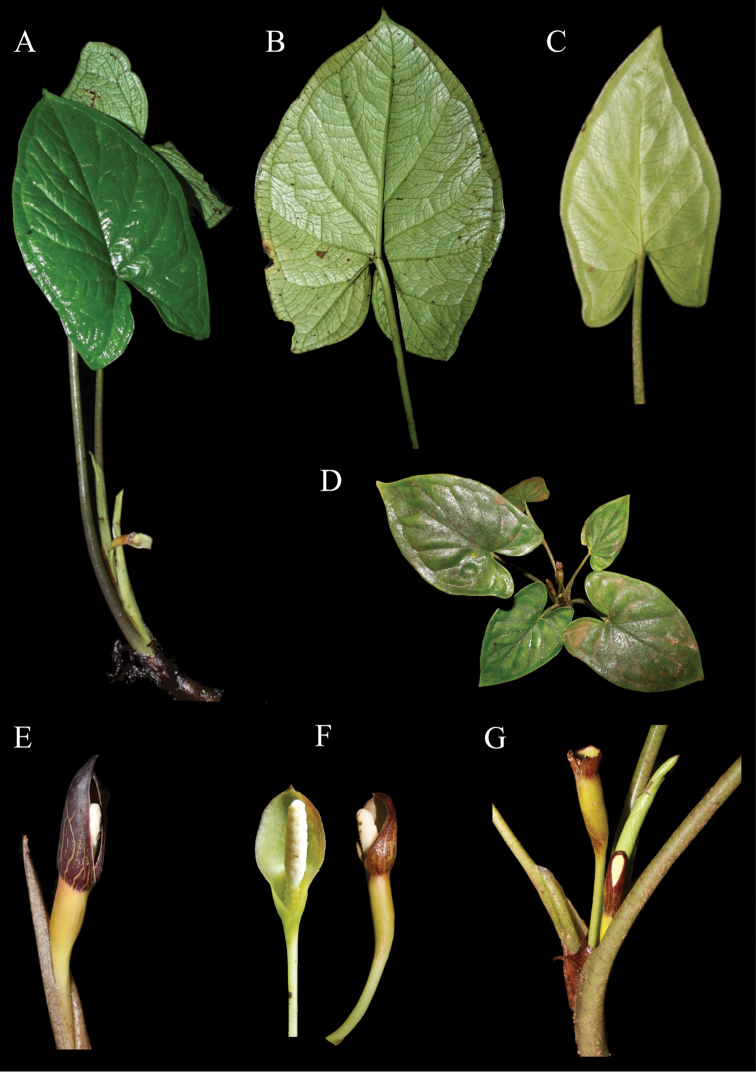
Main morphological characters of *Chlorospatha silverstonei* and *C. minima*. **A, B, E***C. silverstonei***C, D, F, G***C. minima***A** adult plant **B** abaxial blade surface **C** abaxial blade surface **D** adult plant **E** cataphyll and inflorescence in post anthesis, note spathe acuminate and longer than the spadix **F** Inflorescence in anthesis (right) and post-anthesis (left) **G** shoot, showing young infructescence with deciduous spathe blade and young inflorescence.

*Chlorospatha minima* belongs to section Orientales, characterized by having a stylar region lacking a mantle (Fig. 2B). However, all species in section Orientales, as recognized by Croat and Hannon (2015), are endemic to the eastern slopes of the Ecuadorian Andes, whereas *C. minima* and *C. silverstonei* sp. nov. (also described here) are endemic to the western slopes of the Colombian Andes. *Chlorospatha minima* is similar to *C. silverstonei* (see discussion under this species), *C. longipoda* (K.Krause) Madison, *C. hannoniae* Croat, and *C. boosii* Croat & L.P.Hannon, but it differs from these four species in having an overall smaller size, less than 30 cm tall (vs. 30–60 cm). Additionally, *C. minima* is the only species with smooth adaxial leaf surface (vs. quilted or bullate) (Fig. 3), cataphylls of inflorescence not visible outside the petiole sheath, and 1 to 2 inflorescences per axil (vs. 1 to 7) (Table 1).

*Chlorospatha minima* differs from *C. longipoda* in having leaves broadly triangular-ovate vs. narrowly ovate or ovate-elliptic, base of blade slightly hastate vs. subcordate to subsagittate, and (6)20–24 seeds per berry (vs. 7–8). It differs from *C. hannoniae* in having leaves weakly hastate at base vs. sagittate or subsagittate, apex of spathe apiculate vs. cuspidate, and erect spadix vs. slightly curving forward. Finally, *C. minima* differs from *C. boosii* in having 2–5 leaves that are held erect (vs. 8 to 12 leaves) (Table 1). Also, in both species described here, we observed several layers of apparently dead cells on the abaxial surface forming a reticulum visible on dried specimens (Fig. 2F–G, 2M–N). This is not mentioned on the description of other species of section Orientales; therefore this could be a potential diagnostic character.

**Table 1.** Morphological comparison of *Chlorospatha minima*, *C. silverstonei*, *C. longipoda*, *C. hannoniae* and *C. boosii*.

	*C. minima*	*C. silverstonei*	*C. longipoda*	*C. hannoniae*	*C. boosii*
**Plant size**	10–25 (–30 cm)	30–60 cm	40 cm	50 cm	30–50 cm
**Bulbils**	Absent	absent	absent	Present	present
**Cataphylls**	quickly deciduous	persisting ± intact	Remnants of old cataphylls persisting ± intact to semi-intact.	ultimately deciduous	quickly deciduous
**Number of leaves**	2–5	1–3	3–5	8–14	8–12
**Leaf shape**	broadly triangular-ovate	broadly ovate to rounded	narrowly ovate or ovate-elliptic	ovate-cordate, occasionally broadly subtriangular	ovate (occasionally subsagittate in juvenile plants)
**Base of blade**	inaequilateral, slightly hastate	cordate, rarely slightly hastate	subcordate to subsagittate	sagittate or subsagittate	cordate-subcordate
**Apex of blade**	acuminate	acuminate to cuspidate, almost always mucronate at apex	weakly to moderately acuminate to bluntly acute or apiculate at apex	weakly acuminate to apiculate at apex	weakly acuminate or apiculate at apex
**Blade size**	5.8–14.2(–16.3) × 2.6–10.1 cm	9.3–27.7 × 4.9–17.7(–21.2) cm	(10.0–)15.5–21.5 × (3.5–)5.0–13.0 cm	16.0–20.5 × 10.0–15.5 cm	(16.0–)19.0–27.5 × (7.0–)13.0–19.5 cm
**Adaxial leaf surface**	Smooth	quilted	quilted	broadly quilted and sub-bullate	broadly quilted
**Abaxial leaf surface**	broadly reticulate, with dead cells layers	reticulate, with dead cells layers	reticulate, narrowly colliculate along all venation	reticulate, narrowly minutely colliculate along all venation	reticulate, narrowly minutely colliculate along all venation
**Diameter of reticulum on abaxial surface of the leaf**	0.3–0.4 mm diam.	0.2–0.3 mm diam.	present, not seen	present, not seen	present, not seen
**Secondary and minor venation on lower surface**	sunken	prominulous	convex or moderately to narrowly round-raised	prominulous	slightly raised
**Indument of veins in lower surface**	Absent	present	unknown	unknown	unknown
**Number of acroscopic veins**	1–2(–3)	1–3(–4)	2–3	unknown	2–3
**Number of basiscopic veins**	2–3(–4)	3–4(–5)	2–3	unknown	3 to 4
**Number of primary lateral veins**	3(–4) pairs	2–4(–6) pairs	4–6 pairs	(3–)4 pairs	3–4 pairs
**Angle of primary lateral veins**	30°–60°(70°)	30°–70°(90°)	17°–45°	45°–65°	25°–55°
**Number of collective veins**	3	2	2–3(–4)	2(–3)	3
**Inflorescences per axil**	1 to 2	1 to 4	1 to 6	3 to 5	4 to 7
**Cataphylls of inflorescence**	not visible outside petiole sheath	visible outside petiole sheath	visible outside petiole sheath	visible outside petiole sheath	visible outside petiole sheath
**Portion of the spathe exceeding the spadix**	6.6–7 mm	4.3–5.0(–25.7) mm	10.0–35.0 mm	7.0–10.0 mm	20.0–40.0(60.0) mm
**Spadix position**	Erect	erect	erect, occasionally curving forward at anthesis	slightly curving forward	erect
**Spadix length**	20.4–22.8 mm	25.0–37.3 mm	(33.0–)43.0–53.0 mm	38.0–50.0 mm	56.0–69.0 mm
**Length of spadix adnate to spathe**	2.3–3.1 mm	3.3–5.1 mm	6.0–8.0 mm	2.0–3.0 mm	3.0–7.0 mm
**Proportion of adnate portion**	1/3	1/3	1/2	1/4 or less	1/4 to ca. 1/2
**Pistillate portion length**	8.3–8.6 × 2.0 mm	8.7–16.1 × 2.5–2.9 mm	(7.0–)10.0–18.0 × 2.0–3.5 mm	7.0–12.0 × 2.0–4.0 mm	10.0–15.0 × 3.0–3.5 mm
**Staminate portion length**	11.2–12.9 × 2.8–3.0 mm	13.6–19.6 × 4.1–4.4 mm	20.0–30.0 × 2.0–3.0 mm	25.0–35.0 × 3.0–3.5 mm	34.0–45.0 × 3.5–4.0 mm
**Sterile portion length**	2.2–2.5 × 2.7 mm	3.1–3.9 × 3.7–4.3 mm	4.0–8.0 × ca. 2.0 mm	7.0–12.0 × 2.0–3.0 mm	5.0–9.0 × 2.5–3.0 mm
**Border of sterile flowers**	Straight	irregular	irregular	irregular	irregular
**Color of spathe in fruit**	Brown	black	entirely green or occasionally purple-tinged	unknown	unknown
**Seeds per berry**	(6–)20 to 24	6 to 20	7 to 8	unknown	unknown

##### Specimens examined.

**COLOMBIA**. **Valle del Cauca**: municipio Dagua, corregimiento El Queremal, 3°33'45.6"N, 76°45'27.1"W, 1159 m, 17 Mar 2018, Zuluaga et al. 2328 (CUVC!).

#### Chlorospatha silverstonei

Taxon classificationPlantaeAlismatalesAraceae

Zuluaga & Muñoz-Castillo sp. nov.

02B65A3E-9D72-5DCF-84A0-1B56727F5E50

urn:lsid:ipni.org:names:77202844-1

Figs 1 , 2 , 3 , 5

##### Type.

COLOMBIA. Valle del Cauca: municipio El Cairo, Reserva Natural de la Sociedad Civil “Cerro El Inglés”, camino al límite departamental entre Valle y Chocó. 4°44'13.3"N, 76°18'7.7"W, 2120–2230 m, 8 Oct 2017, A. Zuluaga & M.E. Cardona 1946 **(holotype**: CUVC!; **isotypes**: COL!, MO!)

##### Diagnosis.

*Chlorospatha silverstonei* can be distinguished from the other species in section Orientales by having 1–3 leaves per plant, an overall larger size (30–60 cm tall) and a small spadix (25–37.3 mm long). Additionally, it differs from *C. minima* sp. nov., the other species in this section from the western slopes of the Colombian Andes, in having two collective veins (vs. three in *C. minima* sp. nov.), the primary lateral and minor veins with scale like indument on the abaxial surface (vs. glabrous).

Terrestrial herb, 30–60 cm tall; stem decumbent, with remnants of cataphylls persisting ± intact; internodes 5.2–20.2 × 6.8–12.5 mm, drying matte, dark brown; cataphylls brownish green, (3.1–)4.4–8.5 cm long, acuminate at apex, drying faintly glossy, reddish brown. Leaves 1 to 3, erect-spreading; petioles 15.2–46.8(–52.4) cm long, free portion of the petiole 1.72–7.18 mm diam. midway, fleshy, glabrous, semiglossy, irregularly dark purple-mottled with longitudinal dark purple lines, drying dark brown to black, sheathed 2.7–11.5(–15.0) cm, less than 2/5 of its total length; sheath decurrent on to the petiole apex; blades broadly ovate to rounded, glabrous, conspicuously bicolor, 9.3–27.7 × 4.9–17.7(–21.2) cm, 1.2 to 2.3 times longer than wide, cordate at base, rarely slightly hastate, acuminate to cuspidate at apex, almost always mucronate at apex, usually slightly broader across anterior lobe than at base, distance tip to tip across posterior lobes 3.1–13.9(–20.5) cm, not constricted at petiole insertion; adaxial surface quilted, glossy, drying brownish green; abaxial surface reticulate, glossy, drying green to yellow-green, with several layers of cells forming a reticulum, 0.2 to 0.3 mm diam.; anterior lobe 7.2–17.4(–18.5) × 4.9–17.7(–21.2) cm, 0.8 to 1.7(–2.0) times longer than wide, 1.5 to 3.6(–4.0) times longer than posterior lobes, broader near petiole insertion, ± symmetrical; posterior lobes directed toward base, 2.1–9.6 × 1.7–8.3 cm, 0.68 to 1.45 times longer than wide, rounded to obtuse at apex, weakly broader at base, slightly inequilateral, the inner side narrower, sinus spathulate to ovate; midrib and major venation narrowly sunken adaxially, round-raised, drying ± flattened and usually darker than surface abaxially; primary lateral veins 2 to 4 per side, rarely 5–6, arising at 30°–70°, rarely 90°, straight to weakly curved towards the margin; secondary and minor veins darker than the surface, prominulous, forming a conspicuous reticulum abaxially, more visible when dried, the primary lateral and minor veins with scale-like indumentum abaxially, only visible in dried material under the microscope; collective veins 2, the outermost arising from the first and second basiscopic veins, ± parallel to margin, the innermost arising from the third basiscopic vein; basal veins coalescent into a prominent posterior rib, 1–3(–4) acroscopic veins, 3–4(–5) basiscopic veins. Inflorescences erect (all measurements made from spirit material), 1 to 4 per axil; cataphylls of the inflorescence visible outside the petiole sheath, irregularly dark purple-mottled, drying dark brown to black; peduncle 42.8–53.1 (–112.7) × 0.8–1.3 mm, held within the sheath, drying dark brown to black; spathe erect, 32.2–62.9 mm long, acuminate at apex, 4.3–5.0(–25.7) mm longer than spadix (1.2 to 1.7 times longer than spadix); spathe tube pale green on outer surface, rarely maroon-tinged, 8.7–23.3 × 5.0–6.3 mm, drying dark brown to black on outer surface; spathe blade maroon-tinged, with green veins on outer surface, green on inner surface, 18.7–39.7 mm long, drying dark brown or black, erect after anthesis, marcescent in fruit; spadix erect, 25.0–37.3 mm long, sessile, adnate basally to the spathe for 3.3–5.1 mm (ca. 1/3 of the length of the pistillate portion); pistillate portion light green, 8.7–16.1 × 2.5–2.9 mm; pistils coherent, ca. 1.0 mm diam.; stigma light green, sessile, ca. 0.3 mm diam.; fertile staminate portion white, 13.6–19.6 × 4.1–4.4 mm, slightly conical, rounded at apex, drying whitish brown; synandria ca. 1.1 mm diam., coherent; sterile portion white, 3.1–3.9 × 3.7–4.3 mm, wider at apex, drying whitish brown; sterile flowers with irregular borders, ca. 1.4 mm (viewed from above). Infructescence erect or pendent, brown, 18.5–32.6 × 4.8–10.3 mm, drying dark brown on outer surface; berries drying pale tan; seeds white, 6 to 20 per berry, 1.5–2.0 × 0.9–1.2 mm, ovoid to ellipsoid, longitudinally striate, minutely strophiolate, drying brown.

**Figure 4.**
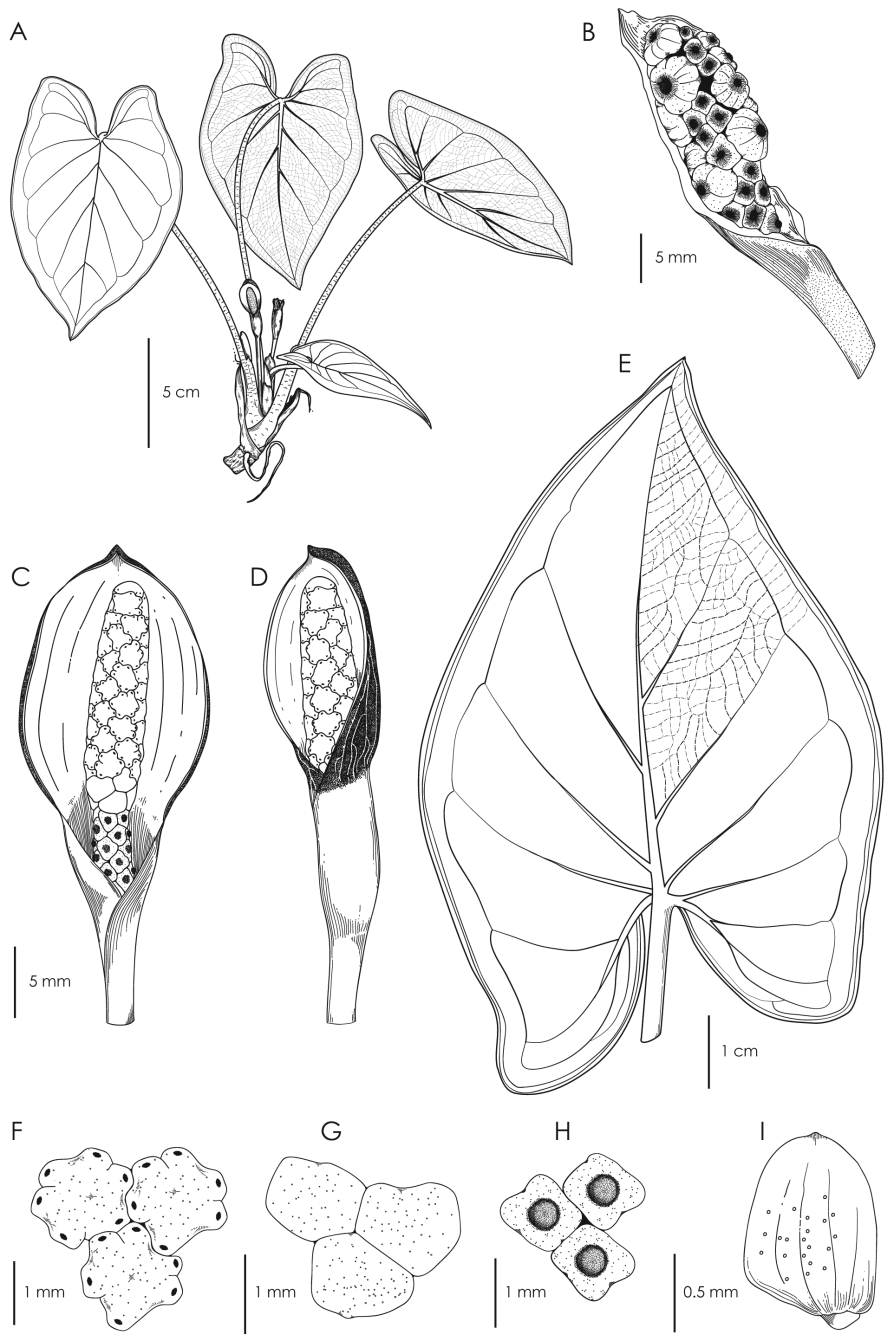
Illustration of *Chlorospatha minima***A** adult plant with inflorescence **B** infructescence **C** inflorescence at anthesis **D** inflorescence on post-anthesis **E** abaxial surface of leaf blade; note reticulate venation and collective veins **F** upper view of male flowers **G** upper view of sterile flowers **H** upper view of female flowers **I** seed. (Drawn by Eileen Muñoz from the holotype A. Zuluaga et al. 1645).

**Figure 5.**
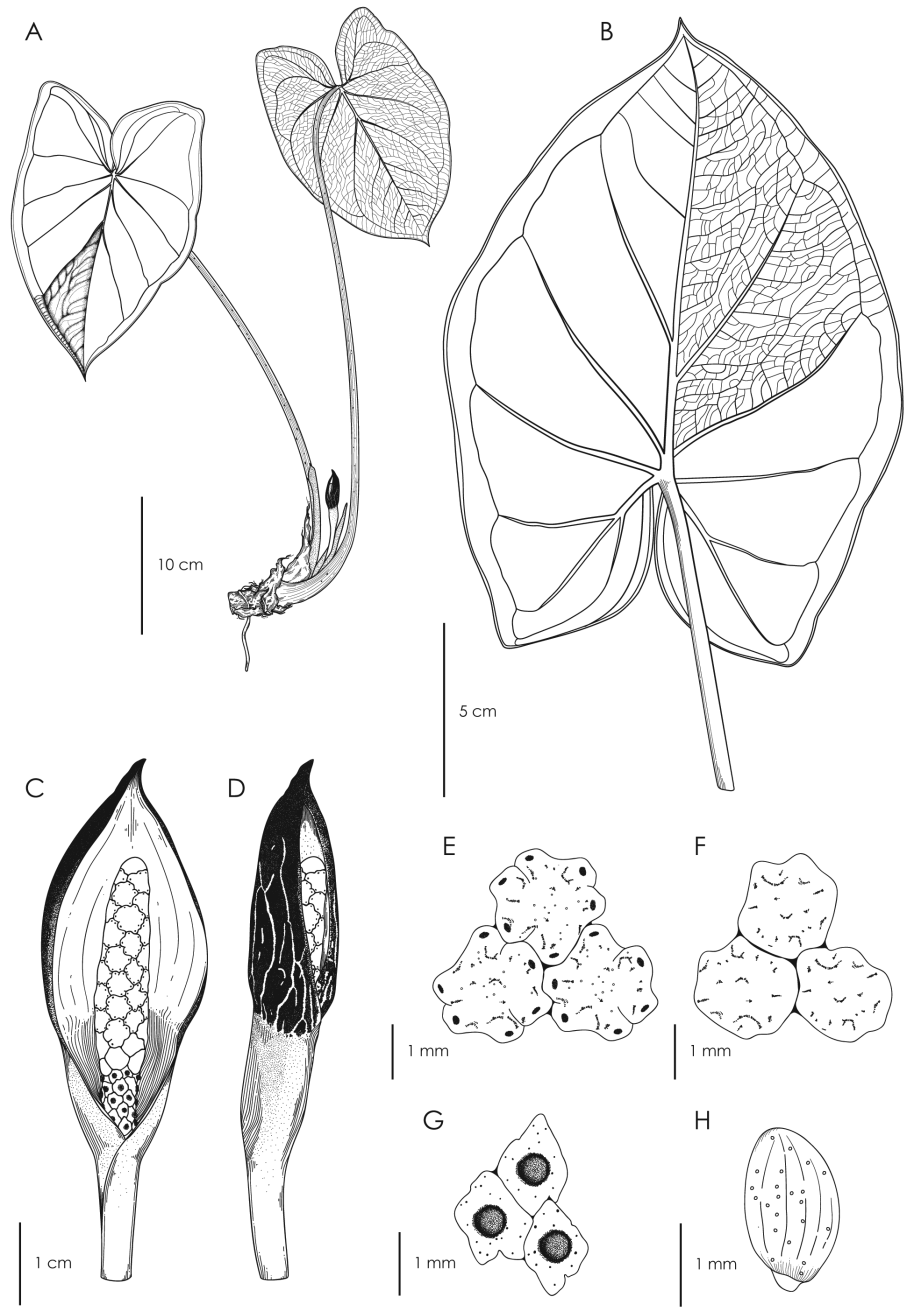
Illustration of *Chlorospatha silverstonei***A** adult plant with inflorescence **B** abaxial surface of leaf blade; note reticulate venation and collective veins **C** Inflorescence in female anthesis **D** Inflorescence in post-anthesis **E** upper view of male flowers **F** upper view of sterile flowers **G** upper view of female flowers **H** seed. (Drawn by Eileen Muñoz from the holotype A. Zuluaga et al. 1946).

##### Etimology.

*Chlorospatha silverstonei* is named in honor of Dr. Phillip Silverstone-Sopkin (1939–2018), an American botanist who lived and worked in Colombia for 39 years. He was a faculty member at Universidad del Valle until 2014 and an *ad Honorem* professor since 2015. Additionally, he was the director of the herbarium Luis Sigifredo Espinal Tascon at the same University for 17 years. Dr. Silverstone-Sopkin collected more than 13000 botanical specimens, especially from the department of Valle del Cauca, and carried out several explorations in the region where this species was found.

##### Distribution and ecology.

*Chlorospatha silverstonei* is endemic to the western slopes of the Colombian Andes, along the border between the departments of Valle del Cauca and Chocó. It grows in cloud forests between 1900 and 2300 m. It has been collected in two natural reserves, “Cerro El Inglés” and “Alto Galapagos” (Fig. 1), where it has been found widespread in the dark understory, with high humidity and, sometimes, flooded ground. This species has been recorded flowering in October and January. Information about pollination is still lacking but we observed individuals of a species of Brachonidae (Hymenoptera) visiting the inflorescence during female anthesis.

##### Conservation status.

*Chlorospatha silverstonei* has been found in two localities along the Serranía de los Paraguas mountain range, with an estimated extent of occurrence larger than 38000 km^2^. In these two localities there are several populations of this species with abundant individuals; therefore, *C. silverstonei* is preliminary categorized as Least Concern (LC), according to the IUCN criteria (IUCN 2012, 2017).

##### Discussion.

*Chlorospatha silverstonei* is similar to *C. minima*, *C. longipoda*, *C. hannoniae*, and *C. boosii*, but it differs from these four species in having fewer leaves (1–3 vs. 2–14) (Table 1). *Chlorospatha silverstonei* differs from *C. minima* in having 1–3 leaves (vs. 2–5 in *C. minima*), longer petioles, 15.2–46.8(–52.4) cm long (vs. 8.3–28.2 cm), that are irregularly dark purple-mottled with longitudinal dark purple lines (vs. green with darker transverse markings), blade broadly ovate to rounded (vs. broadly triangular-ovate), two collective veins (vs. three), fertile staminate portion slightly conical (vs. cylindrical), infructescence 18.5–32.6 × 4.8–10.3 mm (vs. 25.5 × 8.0 mm) and seeds 1.5–2.0 × 0.9–1.2 mm (vs. 1.2–1.5 × 0.7–0.9 mm). Finally, *C. silverstonei* differs from *C. hannoniae* and *C. boosii* in having cataphylls persisting ± intact (vs. cataphylls ultimately deciduous or quickly deciduous) and the absence of bulbils.

##### Specimens examined.

**COLOMBIA. Chocó**: municipio Sipí, Reserva Natural Cerro El Inglés, debajo del sitio Santicos, 4°45'22.0"N, 76°18'12.9"W, 2000 m, 17 Oct 2016, A. Zuluaga et al. 1321 (CUVC!). **Valle del Cauca**: municipio El Cairo, Reserva Natural Cerro El Inglés, camino desde la divisoria de aguas hasta la cabaña de investigadores, 4°44'23.9"N, 76°18'15.0"W, 2100–2200 m, 22 Jan 2016, A. Zuluaga et al. 946 (CUVC!); camino a Los Santicos, 4°45'15.5"N, 76°18'02.3"W, 2250 m, 17 Oct 2016, A. Zuluaga et al. 1305 (CUVC!); reserva natural Alto Galapagos, near to the border Chocó-Valle del Cauca, 2018, A. Zuluaga et al. (CUVC!).

## Supplementary Material

XML Treatment for Chlorospatha minima

XML Treatment for Chlorospatha silverstonei
